# Fasting diets: what are the impacts on eating behaviors, sleep, mood, and well-being?

**DOI:** 10.3389/fnut.2023.1256101

**Published:** 2024-01-09

**Authors:** Elham Hosseini, Achraf Ammar, Jessica K. Josephson, Deanna L. Gibson, Gholamreza Askari, Nicola L. Bragazzi, Khaled Trabelsi, Wolfgang I. Schöllhorn, Zeinab Mokhtari

**Affiliations:** ^1^Nutrition and Food Security Research Center, Isfahan University of Medical Sciences, Isfahan, Iran; ^2^Department of Training and Movement Science, Institute of Sport Science, Johannes Gutenberg-University Mainz, Mainz, Germany; ^3^High Institute of Sport and Physical Education, University of Sfax, Sfax, Tunisia; ^4^Research Laboratory, Molecular Bases of Human Pathology, LR19ES13, Faculty of Medicine, University of Sfax, Sfax, Tunisia; ^5^Department of Biology, University of British Columbia, Kelowna, BC, Canada; ^6^Faculty of Medicine, University of British Columbia, Kelowna, BC, Canada; ^7^Department of Community Nutrition, School of Nutrition and Food Science, Isfahan University of Medical Sciences, Isfahan, Iran; ^8^Human Nutrition Unit (HNU), Department of Food and Drugs, University of Parma, Parma, Italy

**Keywords:** diet, fasting, mental health, eating behavior, sleep

## Abstract

Fasting diets (FDs) have drawn great attention concerning their contribution to health and disease over the last decade. Despite considerable interest in FDs, the effect of fasting diets on eating behaviors, sleep, and mood-essential components of diet satisfaction and mental health- has not been addressed comprehensively. Understanding the critical role that fasting plays in these elements will open up potential treatment avenues that have not yet been explored. The aim of the present paper was to conduct a comprehensive critical review exploring the effects of fasting on eating behaviors, sleep, and mood. There is currently a lack of clarity regarding which fasting option yields the most advantageous effects, and there is also a scarcity of consistent trials that assess the effects of FDs in a comparable manner. Similarly, the effects and/or treatment options for utilizing FDs to modify eating and sleep behaviors and enhance mood are still poorly understood. Further researches aiming at understanding the impacts of various fasting regimes, providing new insights into the gut-brain axis and offering new treatment avenues for those with resistant anxiety and depression, are warranted. Alteration of eating behaviors can have lasting effects on various physiological parameters. The use of fasting cures can underpin ancient knowledge with scientific evidence to form a new approach to the prevention and treatment of problems associated with co-morbidities or challenges pertaining to eating behaviors. Therefore, a thorough examination of the various fasting regimens and how they impact disease patterns is also warranted.

## Introduction

The use of fasting to impact health outcomes can be traced back to the Hippocratic collection which treated seizures in a man by abstaining from food and drink (1). More recently, Guelpa and Marie (2) and later Höhn et al. (3) tested intermittent fasting as a potential intervention for epilepsy. Fasting diets (FDs) have drawn a great deal of attention among energy-restricted diet options (4) due to their associated effects on reducing serum glucose, depletion of hepatic glycogen, and a shift from glycolysis to ketogenesis. Concurrently, attention has been drawn to its various physical and mental health effects (5) such as altered anxiety behavior and cognition effects (6).

Due to their perceived ease of dietary control and satisfaction, FDs are associated with greater adherence and acceptance compared to common calorie-restricted regimens (7). This perceived dietary control has also been tied to FDs impact on eating behaviors (8). Eating behaviors are traits which have an effect on calorie consumption via food choices as well as decisions about when to start eating, how much to eat, and when to stop eating (9). These traits can be tied to well-being and therefore, understanding the impact of FDs on eating behaviors helps to choose a more appropriate approach in practice.

Mounting evidence shows that FDs confer positive effects on weight control (10) and a beneficial contribution against a wide variety of health outcomes such as metabolic syndrome, type 2 diabetes mellitus (T2DM), cardiovascular diseases, and cancer (11). Moreover, several human and animal studies have reported the anti-aging effects (12) and neurological benefit of FDs. Similarly, the implementation of FDs has been shown to relieve symptoms of many disorders such as Alzheimer’s disease (AD), Parkinson’s disease (PD), multiple sclerosis (MS), epilepsy (13), and stroke (14).

Despite considerable interest in FDs, the effect of fasting diets on eating behaviors, sleep, and mood as critical elements in diet satisfaction and mental health has not been addressed comprehensively. Developing a comprehensive understanding of the role played by fasting on these elements as well as on mental health in general, could open up new avenues for potential treatments that are currently unexplored. The aim of the present paper was to conduct a comprehensive review that critically examines the effects of fasting on eating behaviors, sleep, and mood. A thorough synthesize of the existing literature and available evidence was conducted, and key findings and potential mechanisms underlying the possible benefits of FDs on mental health were highlighted.

As there is substantial variability within types of fasting diets, the first topic of this paper focused on the momentary classification of different fasting diets that will be used in subsequent sections, namely the alternate day fasting (ADF), 5:2 intermittent fasting (IF), periodic fasting, time-restricted feeding (TRF), and religious fasting regimes.

Afterward, the present paper reviewed the impact of different fasting protocols on (i) eating behaviors; (ii) sleep patterns; and (iii) mood and well-being. By comprehensively examining the body of evidence related to these interconnected aspects, we aim to gain insights into the potential benefits or challenges associated with fasting interventions in relation to eating behaviors, mood regulation, and mental well-being, as well as provide insights into potential strategies for improving sleep health and managing sleep disorders.

Lastly, this review emphasizes the potential mechanisms underlying the impacts of FDs on brain and mental health, with a specific focus on the gut microbiome pathway. Through this critical review, we aim to contribute to the existing knowledge base and identify gaps in current research.

### Literature search

For this narrative review, a literature search was conducted on PubMed and Web of Science in May 2023 using a predefined search strategy that combined key words related to various fasting diet regimens and different behavioral and wellbeing related outcomes. Additionally, a hand-search of the citations retrieved from PubMed and Web of Science was performed, and any additional relevant studies were considered for review. No restrictions were applied. The inclusion criteria involved studies that examined the impact of fasting diets on at least one of the outcomes of interest, and these selected studies were then reviewed.

### Fasting diet regimens

Fasting diets have been implemented in different forms. Unlike traditional calorie restriction diets, fasting diets require a minimum of 8 h of fasting. ADF is defined as a restriction of 75% to 100% of the energy needs every other day and consuming the usual diet on the other days. The 5:2 IF which is one of the most popular regimens for FDs, involves fasting for 2 non-consecutive days and eating a habitual diet on the other 5 days of the week. In periodic fasting, people are recommended to limit food intake for 1 or 2 days per week but are allowed to eat their regular diet for 5 or 6 days per week. TRF is characterized by eating abstinence for particular time periods of the day, generally between 8 and 12 h each day. Religious fasting regimes, such as Ramadan, in which individuals must fast from dawn to sunset for a month, are identified as other forms of fasting diets (15, 16).

### Effect of different FDs regimens on eating behaviors

Eating behaviors are determined by various factors, including genetic, biological, and behavioral variables; psychological background; and environmental factors, including social, cultural, economic, and demographic pressures (9). In fact, eating behaviors are strongly linked to obesity and are influenced by weight management approaches. Several dimensions related to eating behavior and dieting have been investigated in the literature. Tacad et al. (17) and Llewellyn et al. (18) have postulated that appetite drives human beings to seek and eat food, a process regulated by homeostatic control of food consumption, hedonistic signals, eating experiences, and the reward system, while others have focused on factors related to stopping eating, including satiety or fullness. Some have considered both sides.

It is suggested that fasting diets can be considered as an alternative to traditional caloric restriction diets. Weight loss through caloric restriction (CR) is a complex issue, particularly during long-term dietary restriction. Often, compensatory responses lead long-term CR subjects to overeat, causing the lost weight to be regained and, in some cases, surpassed (19). Indeed, a considerable increase in appetite and desire to eat after >5% body weight loss has been reported (20, 21). Studies by Varady et al. (22) and Hansen et al. (23) revealed that fasting diets are superior to caloric restriction in mitigating the losses of lean body mass during weight loss, potentially attenuating hyperphagic responses, hunger sensations, and weight regain. Indeed, satiety improving and/or hunger-weakening treatments are suggested as potentially successful weight management strategies (23). However, results are not clear-cut, as other studies have reported similar effects on body composition from both CR and FD (24). Currently, the effects of fasting diets on eating behaviors have been investigated in a few studies with conflicting results (Table 1). Understanding eating behavior modifications followed by FD is necessary to evaluate the effectiveness of this intervention.

**Table 1:** Studies evaluating the effects of fasting diets on eating behaviors.

Study (ref.)	Population	Fasting type & regimen	Time of treatment	Findings
Keenan (25) 2022 Australia	Both sexes BMI range: 22–35 kg/m^2^ Age range: 18–35 y 5:2 diet + Exercise group: *n* = 17 CR diet + Exercise group: *n* = 17	5:2 diet + Exercise: 2 d fasting (restriction of 70% E + TRF between 12 p.m. and 6 p.m.); 5 d feeding (Euenergetic consumption); 3 resistance training sessions/week CR diet + Exercise: 20% E restriction; 3 resistance training sessions/week In both groups: Energy intake = approximately 80% E per day and pro intake ≥1.4 gr/kg/d	3 months	Hunger: ↓ 5:2 diet + Exercise, ↓ CR diet + Exercise Craving: ↔ 5:2 diet + Exercise, ↔ CR diet + Exercise
Kalam (26) 2021 United States	Both sexes BMI range: 30–50 kg/m^2^ Age range: 18–65 y *n* = 31	ADF: consuming 600 kcal on fasting days and usual diets on feed days; 30 E% carbohydrates; 35 E% protein and 35 E% fat	6 months	↔ Fasting and postprandial hunger ↔ Fasting and postprandial fullness
Teong (27) 2021 Australia	Women BMI range: 25–42 kg/m^2^ Age range: 30–70 y ADF group: *n* = 22 CR group: *n* = 23	ADF group: consuming 30% E requirement as breakfast at 8 a.m. on fasting days and fasted for 24 h and usual diets on feed days CR: consuming 70% E requirement	2 months	Dietary restraint, disinhibition or hunger: ↔ Both groups Depression, anxiety or stress, and sleep quality: ↔ Both groups
Oustric (28) 2021 United Kingdom	Women BMI 25.0–34.9 kg/m^2^ Age range: 18–55 y ADF group: *n* = 19 CR group: *n* = 18	ADF group: consuming 30% E requirement and usual diets on feed days CR: consuming 75% E requirements	3 months	Liking for high fat-sweet, high-fat-savory, low-fat-sweet and low-fat-savory: ↓ ADF, ↓ CR Wanting for high fat-sweet, high-fat-savory, low-fat-sweet and low-fat-savory: ↔ ADF, ↔ CR
Beaulieu (29) 2020 United States	Women BMI range: 25–34.9 kg/m^2^ Age range: 18–55 y ADF group: *n* = 24 CR group: *n* = 22	ADF group: consuming 25% E requirement on fasting days and usual diets on feed days CR group: consumption of 75% E needs per day	Reaching to ≥5% weight loss, in ADF group: about 67 days, in CR group: about 57 days	Hunger: ↓ ADF, ↓ CR Satiety: ↔ ADF, ↔ CR Dietary restraint: ↓ ADF, ↓ CR Desire to eat: ↓ ADF, ↓ CR Disinhibition: ↑ ADF, ↓ CR Susceptibility to hunger: ↓ ADF, ↓ CR Binge eating scores: ↓ ADF, ↓ CR Craving for sweet foods: ↓ ADF, ↓ CR Craving for savory foods: ↑ ADF, ↓ CR Food reward (Liking and wanting for high-fat relative to low-fat): ↔ ADF, ↔ CR
Ravussin (30) 2019 United States	Both sexes BMI range: 25–35 kg/m^2^ Age range: 20–45 y *n* = 10	TRF: eat between 8:00–14:00; 6 h daily eating period for 4 days and then to cross over to the other arm after a 3.5–5 weeks washout period Control group: eat between 8:00–20:00 h; 12 h daily eating period	4 days	Hunger: ↔ TRF, ↓ Control group Fullness: ↑ TRF, ↔ Control group Desire to eat: ↓ TRF, ↓ Control group
Hutchison (31) 2019 Australia	Men Mean BMI: 33.9 ± 0.8 kg/m^2^ Age range: 30–70 y *n* = 15	Early TRF: time restricted eating from 8 a.m. to 5 p.m. Delayed TRF: time restricted eating from 12 p.m. to 9 p.m., separated by a 2 weeks washout phase	7 days	Hunger, fullness, and desire to eat: ↔Early TRF, ↔Delayed TRF
Sutton (32) 2018 United States	Men with prediabetes BMI: 32.2 ± 4.4 kg/m^2^ Mean age = 56 ± 9 *n* = 8	TRF: 6 h feeding period, with dinner before 3 p.m., with 18 h of daily fasting. Control group:12 h feeding period	5 weeks	Desire to eat in the evening ↓ TRF Fullness in the evening ↑ TRF
Kroeger (33) 2018 United States	Both sexes BMI range: 25–40 kg/m^2^ Age range: 18–65 y ADF group: *n* = 34 ADF with ≥5% weight-loss: *n* = 14 ADF with weight-loss <5%: *n* = 20 CR group: *n* = 35 CR with ≥5% weight-loss: *n* = 14 CR with weight-loss <5%: *n* = 21	Weight loss phase ADF group: consuming 25% E requirement on fasting days and 125% E requirement on feed days CR group: consumption of 75% E needs per day Weight maintenance phase ADF group: consuming 50% E requirement on fasting days and 150% E requirement on feed days CR group: consumption of 100% E needs per day	12 months 6 months weight loss phase and 6 months weight maintenance phase	Hunger: ↓ ADF with ≥5% weight-loss after/before ↔ ADF with weight-loss <5% after/before ↔ CR group after/before Fullness: ↓ ADF with ≥5% weight-loss after/before ↔ ADF with weight-loss <5% after/before ↔ CR group after/before Restrained eating and self-efficacy: ↔ in all groups
Coutinho (34) 2018 Norway	Both sexes BMI range: 30 < BMI < 40 kg/m^2^ Age range: 18–65 y ADF group: *n* = 18 CR group: *n* = 17	ADF group: consuming 550 and 660 kcal/day for women and men, respectively, on fasting days and habitual diet on feed days CR group: conventional calorie diet	3 months	Hunger, Fullness, Desire to eat: ↔ Both groups
Hoddy (35) 2016 Hoddy (36) 2015 United States	Both sexes BMI range: 30–39.9 kg/m^2^ Age range: 25–65 y *n* = 59	ADF group: consuming 25% E requirement on fasting days and habitual diets on feeding days	2 months	Hunger:↔ Fullness:↑ Restrictive eating:↑ Avoidance of forbidden foods:↔
Bhutani (37) 2013 United States	Women BMI range: 30–39.9 kg/m^2^ Age range: 25–65 y ADF group: *n* = 25 Exercise group: *n* = 24 Combination (ADF + exercise): *n* = 18 Control: *n* = 16	ADF group: consuming 25% E requirement on fasting days + TRF between 12 p.m. and 2 p.m. and habitual diets on feeding days Exercise group: moderate intensity exercise program 3 times/week Combination group (ADF + Exercise): consuming 25% of energy requirement on fasting days and habitual diets on feeding days + TRF between 12 p.m. and 2 p.m. Control group: habitual diet	3 months	Hunger: ↓ ADF ↔ Combination Satisfaction and fullness: ↑ ADF ↔ Combination Restrained eating score: ↑ ADF, ↑ Combination ↔ Exercise, ↔ Control Uncontrolled eating score: ↓ ADF, ↓ Combination ↔ Exercise, ↔ Control Emotional eating score: ↔ ADF, ↓ Combination ↔ Exercise, ↔ Control
Varady (38) 2013 United States	Both sexes BMI range: 20–29.9 kg/m^2^ Age range: 35–65 y ADF group: *n* = 16 Control: *n* = 15	ADF group: consuming 25% E requirement on fasting days + TRF between 12 p.m. and 2 p.m. and habitual diets on feeding days Control group: habitual diet	3 months	Satisfaction ↑ ADF group, ↔ Control group Fullness ↑ ADF group, ↔ Control group
Moreno-Domínguez (39) 2012 Spain	Women Healthy women: *n* = 20 Bulimia nervosa participants: *n* = 21	Fating for 20 h	20 h	Food cravings ↑ Both groups
Klempel (40) 2010 United States	Both sexes BMI range: 30–39.9 kg/m^2^ Age range: 35–65 y *n* = 16	ADF group: consuming 25% of energy requirement on fasting days and habitual diets on feeding days	2 months	Hunger: ↑ During the first week ↓ The rest of the trial Satisfaction: ↓ During the first week ↑ During the last 4 weeks of the study. Fullness: ↓ During the entire 8-week intervention
Johnson (41) 2007 United States	Both sexes Asthma patients BMI >30 kg/m^2^ *n* = 10	ADF group: consuming 320 calories in women and men 380 calories in men on fasting days and habitual diets on feeding days	2 months	Hunger: ↔
Heilbronn (42) 2005 United States	Both sexes BMI range: 20–30 kg/m^2^ Age range: 25–53 y *n* = 16	ADF group: 24 h fasting on fasting days and habitual diet on feed days	22 days	Hunger: ↓ Fullness: ↑
Finch (43) 1998 United Kingdom	Both sexes Age range: 13–63 y *n* = 41	Fasting hours were about 13–15 h/d for more than 21 d during the Ramadan	1 month	Hunger: ↑ During the daily fast, ↑ women > men during the earlier days of Ramadan Desire to eat sweet: ↑ women > men

ref, reference; BMI, body mass index; y, year: E%, energy%; CR, calorie restriction; d, day; ADF, alternate day fasting; hr: hours; TRF: time-restricted feeding; ↑, increased; ↓, decreased; ↔, without change.

In a clinical trial by Klempel et al. (40), a 2 months-long modified alternate day feeding (ADF) program resulted in a weight loss of about 4.6%. To accomplish this, the trial restricted 75% of calorie needs over the fasting day and the intake of a habitual diet during the non-fasting day. Food intake was restricted to a 2 h window; between 12 and 2 p.m. Habituation to this regimen resulted in a decrease in feelings of hunger on fast days after 2 weeks of diet. ADF was also not associated with an increased hyperphagic response on feast days. However, the feeling of fullness stayed low throughout the intervention. This study was limited, however, due to its before-and-after design. Furthermore, perceived hunger, satisfaction with diet, and fullness feelings were measured by validated visual analog scale (VAS) scales only at pre-bedtime. Therefore, they might not reflect these feelings throughout the day. Additionally, the authors failed to have a control group; subsequently, these results need to be interpreted with caution.

Findings of a before-after clinical trial by Hoddy et al. (35) indicated that postprandial hunger stayed unchanged and fullness was increased following 4% weight loss by 2 months ADF (25% energy intake “fast day,” alternated with an *ad libitum* intake “feed day”) (35). This was supported by another study (28) that provided evidence that a 3 months ADF (75% energy restriction days and alternating *ad libitum*) and CR (25% daily energy restriction) can result in similar changes in fat free mass without any increase in compensatory responses in appetite following ~5% weight loss in women with overweight/obesity. Additionally, a decline in hunger and an improvement in eating behavior traits were reported. Similar findings by Varady et al. (38) indicated that in response to 3 months of ADF, there was a 6.5% weight loss compared to the control group in normal-weight and overweight subjects. Besides, increased fullness was found after this intervention (38). Moreover, a decline in hunger was reported after 2%–6% weight loss following an isocaloric 5:2 intermittent fasting (IF) diet as well as a CR diet, both combined with resistance training for 3 months, suggesting that these interventions are well accepted in the medium term (44). Although there is an obvious dissimilarity in hunger between fast and feast days in the 5:2 IF group, there is also a compounding issue of exercise and its effects on eating behaviors. Hunger was disproportionate across fasting/feeding days and was reportedly significantly higher on fasting days than feeding days (25, 44).

Only one clinical trial (33) evaluated the long-term impact of fasting diets on eating behaviors, and it indicated that ADF was not superior to CR with regard to hunger and fullness. Therefore, it seems that short-term alternate-day fasting diet interventions can cause decreases in hunger, although the prolonged beneficial effects of FD are questionable. Long-term studies, however, have not been as conclusive. The findings from short-term studies should not be extrapolated to longer periods, as it remains uncertain how robust these results are over extended durations. This underscores the necessity for additional research to investigate the long-term effects of fasting diets on eating behaviors. A study by Sumithran et al. (45) indicated that greater weight loss (≥8%) may be needed to elicit appetite-related eating behavior compensation (45). However, Coutinho et al. (34) uncovered that the isocaloric diet can induce 12.5% weight loss either by 3 months ADF (intake of 550 and 660 kcal/day for women and men, respectively, over the fasting day and a diet matching energy requirement for achieving a 33% energy restriction during non-fasting day) or CR diet (restriction of 33% of calorie need). Isocaloric dieting may not be associated with changes in fasting or postprandial appetite. Furthermore, hunger was found unchanged in obese adults with moderate asthma following ADF, with an average of 8% weight loss compared to the baseline (41). The inconclusive findings within the literature can be attributed to differences in study designs, heterogeneity of subjects, inadequate sample size, and length of interventions. Firstly, regarding individual differences in the study, it was observed that young individuals and women were more susceptible to show compensatory responses such as overeating and disordered eating behaviors when engaging in fasting diets compared to middle-aged subjects (46, 47). Moreover, variations in appetite and hunger regulation between subjects may serve as mediators in the relationship between fasting and eating behaviors (48). Secondly, differences in duration of interventions may contribute to the variability in results. In shorter periods of fasting diets, greater weight loss may lead to a temporary improvement in body image satisfaction and motivations for dieting, influencing eating behaviors (46). However, adhering to a weight loss diet is often challenging over longer periods, potentially impacting eating behaviors. Further studies are needed to clarify this field.

Eating behaviors during Ramadan differ significantly from regular eating patterns. While being observed, the fasting period is from dawn to sunset, lasting for a month. Once broken, people usually consume high-sugar and high-fat meals during the feeding intervals and have altered sleep cycles. Both sexes report a significant difference in levels of hunger at the beginning of Ramadan compared to the end of the month (43). However, women reported a different fasting experience, with a higher level of hunger reported at the beginning of Ramadan depreciating noticeably towards the end of the month. Considerations of who was involved in food preparation are suggested to stimulate hunger, as exposure to food environments may lead to attenuated feelings of hunger (43). The success of eating behavior modification for longer durations of FDs might also be influenced by variations in dietary composition. Particularly, macronutrient composition factors significantly into appetite control (49). Low-carbohydrate diets (LCDs), which focus on higher protein, fat, and restrictive carbohydrate composition, have become popular in recent decades (50). LCDs can be classified into very-low carbohydrate diets (<3%–30% energy intake), low carbohydrate diets (30%–40% energy intake), and moderate-low carbohydrate diets (40%–45% energy intake) (51). LCDs have demonstrated a suppression effect on hunger and desire to eat, which may be partially mediated via ketotic effects on appetite (49).

Few studies have investigated the alterations in psychological outcomes following fasting diets in conjunction with LCDs. A study by Kalam et al. (26) revealed that hunger and fullness remained unchanged after 5% weight loss induced by a 6 months ADF combined with a low-carbohydrate diet (intake of 600 kcal per fasting day and eating a habitual diet the other days; macronutrient composition: 30% calorie from carbohydrates, 35% calorie from protein, and 35% calorie from fat). Subjective appetite indicators might be interpreted with no compensatory responses after significant weight loss, and it might be a promising finding. However, evidence suggests that restricting carbohydrates to less than 10% of total energy may be needed to produce ketosis and suppress hunger (52). Similar to the study by Klempel et al. (40), Kalam et al. (26) do not have an adequate control group, subsequently, these findings need to be interpreted with caution.

### Fasting diets, dietary restraint and disinhibition

To find the psychological impacts of fasting diets, it is necessary to differentiate eating prompted by hunger from eating to garner pleasure (homeostatic or hedonic, respectively). Dietary restraint and disinhibition are classifications of eating behavior traits that serve as important factors in obesity, dieting, and eating disorders (53, 54). Dietary restraint is identified as a tendency for subjects to intentionally limit food intake in order to maintain or lose weight. Disinhibition refers to overeating in response to emotional conditions, situational states, and food stimuli (9, 53). Dietary restraint and disinhibition are considered as dimensions of eating behaviors. Both dietary restraint and disinhibition have been of special interest for predicting weight-loss-intervention outcomes (55–57) as they are mainly correlated to emotional eating and eating with no hunger (9).

There is some evidence relating the effects of fasting diets to dietary restraint and disinhibition. After two and 3 months of ADF (consumes 25% of energy needs on the fast day and *ad libitum* on each feed day), dietary restraint plateaus (36, 37), with no significant change observed after 12 months of ADF (33). Similar enhancements in eating restraint were observed following 3 months of ADF or CR (29).

It should be noted that within eating behaviors, the cognitive restraint factor is divided into two subscales: rigid and flexible control. Rigid control refers to an “all-or-nothing approach to eating, dieting, and weight,” and flexible control refers to a “more graduated approach to eating, dieting, and weight, in which “fattening” foods are eaten in limited quantities without feeling guilty” (58). Fairburn et al. (59) report that eating behavior adaptation to “all or nothing” can lead to unintentional overeating. Beaulieu et al. (29) found that ADF causes greater rigid restraint and CR causes more flexible restraint. Ultimately, flexible control has been associated with better weight management, fewer eating disorders such as binge eating, and lower adiposity (58, 60). Moreover, it is supported by the fact that a higher dietary restraint can serve as a trigger for food craving which is consequently associated with greater uncontrolled eating and further weight gain (61).

Disinhibition is also divided into two components: internal disinhibition (inability to control eating in response to emotional triggers or emotional eating) and external disinhibition (inability to control eating in response to environmental triggers) (57). ADF’s effects on disinhibition have provided mixed results. An increase in disinhibition, regardless of subscales, was reported following 3 months of ADF compared to CR (29). However, in another study, emotional eating remained unchanged after 3 months of an ADF diet compared to a regular diet (37). A recent study by Teong et al. (27) examined fasting for 24 h three times a week for 2 months and its effects on dietary restraint and disinhibition. Ultimately, in overweight and obese women, ADF was not found to be effective at altering either parameter (27). Based on the literature, the majority of studies examining the effects of ADF and dietary restraint and disinhibition have primarily been conducted over short-term durations and yielded inconsistent findings. Previous studies suggest that among various weight loss regimens, interventions prioritizing the reduction of disinhibition, instead of increasing restraint, may be more successful. This preference for targeting disinhibition may be attributed to its association with disordered eating behaviors and weight gain. However, restraint is a complex factor. While it is linked to improved weight loss on one hand, it is also associated with a susceptibility to food cravings and subsequent weight gain following weight reduction (62).

Currently, it is hard to give a concrete recommendation given the lack of long-term studies on the effects of fasting diets on eating behaviors. Given the various subscales of eating behavior and the potential different effects of fasting diets in comparison with traditional CR, further long-term and isocaloric studies are needed to clarify more details in this area.

### Fasting diets and food craving, food reward, and mindful eating

A food craving is defined as a strong desire to intake certain food items that are calorically dense, sweetened, and/or savory but provide little nutritional utility, for example, chocolate (63). Cravings have been suggested as one of the key factors in overeating (61). The intensity and specificity of some foods make food cravings different from physiological hunger sensations. Food cravings can be alleviated only by selective foods, while hunger can be attenuated by all foods.

Few studies have examined fasting diets regarding food cravings. In addition, food craving is a multidimensional eating behavior that includes physiological, cognitive, and emotional components (63). Previous studies have revealed that there is a complex network between food craving, hunger, food deprivation, and negative emotions (63, 64). It seems that short-term specific food restraint may lead to an increase in food cravings, while long-term CR diets may work to reduce them (63) though the drivers for this remain unclear.

A cross-sectional study conducted among students maintaining a normal weight showed a positive association between a fasting diet combined with a low-calorie diet and food cravings (65). Furthermore, a 20 h fasting period was associated with increased food cravings in healthy women (39). Energy restriction of 100% during fasting days has negative effects on subjective states, including, increased craving and irritability in fasting diets. It seems that less strict energy restrictions of 70%–80% on fasting days make this approach more feasible and acceptable (42). A greater craving for savory foods is reported after ADF compared to CR (29). Moreover, cravings showed an increase following 12 weeks of 5:2 fasting diet and CR (44), which can affect long-term adherence to both diets. However, an improvement in craving control was found after 3 months of ADF compared to a control diet (28, 37). In future research, it would be worthwhile to perform continuous measurement of craving during fast and feast days by new technologies, as well as a comparison of the outcomes of the various fasting diet modes related to several dimensions of food craving. Moreover, given the potential impact of menstrual cycles in women on hunger and cravings (66), there is a lack of evidence that, if considered, might be beneficial in finding the consequences of fasting diets.

Food reward consists of two components: liking and wanting. These terms are always used interchangeably. Nevertheless, they are defined separately and produced by two dissimilar psychological processes. “Liking,” which is pleasure to eat, is mainly related to brain hedonic hotspots and is generally assessed by subjective pleasure ratings in adults. “Wanting” is the driving to eat triggered by food cues, and it is mostly generated by mesocorticolimbic circuitry and commonly evaluated by subjective craving ratings in humans (67). As mentioned in the literature review, food reward is modified by various modes of weight loss interventions. Among these treatments, those that led to increased liking for low-calorie foods and decreased liking for high-calorie foods were associated with greater weight loss and fat mass reduction (68).

The only study that has compared the impact of fasting diets and CR diets on food reward showed that modifications of food reward were similar in response to both FD and CR diets. Unchanged wanting and diminished liking for food with various fat content and sweet tastes were found after more than 5% weight loss (28). Previous studies suggested that two dimensions of food reward are influenced by diet and exercise in a different way; diet impacts liking more than wanting, whereas for exercise this might be the opposite. In fact, wanting contributes more to overeating than liking. Exercise can modulate cognitive processes and motivation, which are more related to the wanting process, whereas dietary interventions might concentrate on the relationship with eating externally, and intrinsic motivations would be less noticed (28, 69). Considering the effects of FD on liking and wanting, besides analyzing brain circuits, helps find more effective approaches for sustainable weight management. More studies are needed to examine the effects of various fasting diets on reward for high-fat, low-fat, sweetened, and savory foods. It should be noted that hunger perception is of a complex nature and is influenced by biological, personal, and environmental factors (70). Individual and environmental differences are lacking in the current ways of measuring hunger. Proper consideration of these heterogeneities is needed for methodological consideration.

Mindful eating is considered a rediscovered concept in determining eating behaviors that can be defined as paying attention to the sense and feeling in response to food, differentiating between physiological and emotional hunger, making food choices consciously, and causing more awareness of internal statuses of satiety and hunger (71). The impact of fasting diets on mindfulness states was explored by Schueler et al. (72), revealing no association between fasting diets and mindful eating among college students. However, another study suggested that fasting diets could potentially enhance mindful eating compared to other diets approaches, such as commercial weight loss programs and low-carbohydrate diets (73). Nevertheless, caution is advised in interpreting the findings of these studies, given the potential limitations inherent in their respective study designs.

### Fasting diets: satiety responsiveness

Satiety responsiveness, an area lacking in current research, is suggested to be associated with alterations in eating behavioral traits (74, 75). The satiation quotient, as an index of satiety responsiveness, is estimated by dividing the difference in appetite sensations in response to a test meal by the calorie content of the meal. People can be categorized into low satiety phenotypes, who have lower satiety sensations before and after a meal, and high satiety phenotypes (76). In spite of similar weight loss in individuals with different satiety responsiveness in response to the traditional CR diet, subjects with lower satiety responsiveness may be more prone to overeating due to greater disinhibition and weaker control over food cravings (74, 75, 77). No study has considered different satiety responsiveness at baseline and particularly examined eating behaviors after weight loss following fasting diets. Therefore, taking into account the individual differences in hunger and satiety sensation that can stem from genetic differences, gender, etc. (18, 78, 79) is essential for both clinical practices to implement individualized medicine approaches, and for clinical research to improve the efficiency of clinical trials.

### Fasting diets, sleep, and circadian rhythm

It has already been noted that FD, as a dieting approach in misalignment with circadian rhythm, might affect sleep-wake pattern (80). Sleep is known as a considerable factor contributing to several cardiovascular and metabolic disturbances and mental disorders. In the current literature, however, less attention has been drawn toward the impact of fasting diets on sleep (80). FDs have also been appraised in the context of mental health issues such as mood and well-being as significant factors affecting the quality of life at any age (81). In this part, we will review the evidence from clinical trials and observational studies to obtain a comprehensive outlook on the possible effects of FDs in association with sleep and mood status. Further, we will discuss the mechanisms through which IF could affect these states. A summary of studies evaluating the effects of fasting diets on sleep and mood status is presented in Table 2.

**Table 2:** Studies evaluating the effects of fasting diets on sleep and mood status.

Study (ref.)	Study design	Fasting type & regimen	Subjects & No.	Comparison group & No.	Study duration/time points	Findings
Beaulieu (70) 2021 United Kingdom	RCT	Intermittent energy restriction: restricted energy to 25% on fast days & free food intake on feed days	Overweight or obese women *N* = 24	Control group with continuous energy restriction *N* = 22	Up to 12 weeks	Increased sleep duration on fast days at week 2 and the final week compared to the baseline. Increased sleep duration on fast days compared to feed days at week 2
Currenti (81) 2021 Italy	Cross-sectional	TRF: eating time of 8 h during the last 6 months	Adults from general population *N* = 1,572	No restricted time	—	No effect on sleep quality
Cienfuegos (82) 2022 United States	RCT	TRF: eating time of 4 or 6 h	Obese adults *N* = 35	Control group (no meal timing restrictions) *N* = 14	8 weeks	No effect of either of regimens on sleep quality, sleep patterns (wake time, bed time, sleep duration, and sleep latency), and insomnia severity index
Grant (83) 2017 Australia	RCT	TRF: simulated night shift, eating at night, 12 h fasting during the day	Healthy adults *N* = 5	Control group with no eating at night *N* = 5	4 consecutive days	Increased sleepiness in both groups. Elevated hunger, full feeling, and urge to eat in the subjects with “no eating at night.” Impaired performance in both groups but it was exacerbated in the comparison group
Hutchison (31) 2019 Australia	Crossover RCT	TRF: early condition (eating hours 8 a.m.–5 p.m.); delayed condition (eating hours 12 p.m.–9 p.m.)	Abdominal obese male adults *N* = 8	Control group with *ad libitum* diet for 7 days *N* = 7	7 days	No change of sleep duration compared to the baseline in either TRF condition
Parr (84) 2020 Australia	Pre-post non-randomized RCT	TRF: eating window (10 a.m.–7 p.m.)	Overweight or obese subjects with type 2 diabetes *N* = 19	Baseline with routine diet for 2 weeks	4 weeks	No effect on mental well-being indicators including sleep quality, duration, disturbance, and day-time sleepiness. No significant change was shown for additional indicators including quality of life, depression, anxiety, and stress of patients
Kesztyus (85) 2020 Germany	Before-after RCT	TRF: eating window of 8–9 h and nightly fasting period of 15 to 16 h	Healthy subjects *N* = 61 Abdominal obese subjects *N* = 38	Baseline	3 months	Increased HRQoL and sleep quality but no alteration of sleep duration
Kalam (86) 2021 United States	Pre-post non-randomized RCT	ADF: 600 kcal “fast day”; *ad libitum* diet “non-fast day” combined with restricted carbohydrate intake to 30%	Obese pre- or postmenopausal women *N* = 31	Baseline period with usual diet and exercise routines for 1 month	Weight loss period: months 0–3 Weight maintenance period: months 3–6	In comparison to the baseline no change in sleep quality, ISI, wake time, bed time, and sleep duration was found by month 3 or 6
Teong (27) 2021 Australia	Parallel RCT	Calorie-restricted (CR) diet versus an IF diet IF, prescribed at 70% calculated energy requirements (CER) 3 times a week (fasting days) and 100% of CER on non-fasting days; CR, prescribed at 70% of CER	Overweight or obese but healthy women *N* (IF) = 22 *N* (CR) = 23	Baseline	8 weeks	No significant difference of mean changes regarding sleep quality, quality of life, mood status, and cognition was observed when comparing IF versus CR
BaHammam (87) 2003 Saudi Arabia	Cross-sectional	Ramadan fasting (RF): abstinence from all food or drink from dawn to sunset	Healthy medical students *N* = 56	Baseline 1 week before Ramadan with normal dietary routine	4 time points: Week before Ramadan, the first week, second week, and third week of Ramadan	A greater daytime sleepiness during Ramadan
Almeneessier (88) 2019 Saudi Arabia	Cross-sectional	RF: abstinence from all food or drink from dawn to sunset	Healthy male volunteers *N* = 8	Baseline 2 weeks before Ramadan with routine diet	2 time points: 2 weeks before Ramadan, the first 3 weeks of Ramadan	No difference in sleep time compared to the baseline. Delayed bed time and wake time in Ramadan
BaHammam (89) 2013 Saudi Arabia	Cross-sectional	RF: abstinence from all food or drink from dawn to sunset	Healthy male volunteers *N* = 8	Baseline 1 week before Ramadan with normal dietary routine	2 time points: 1 week before Ramadan, the first 2 weeks of Ramadan	Delayed bed time and wake time during Ramadan. A significant decrease in total sleep time but no change in daytime sleepiness
Alghamdi (90) 2020 Saudi Arabia	Cross-sectional	RF: abstinence from all food or drink from dawn to sunset	Individuals with Type 2 Diabetes *N* = 36	Seven consecutive days 2 weeks after the end of Ramadan	2 time points: 1 week after Ramadan, 1 week in the middle of Ramadan	A decrease in daily total sleep time and night sleep time in the middle of Ramadan
Bener (91) 2021 Turkey	Cross-sectional	RF: abstinence from all food or drink from dawn to sunset	Patients with hypertension *N* = 1,118	Baseline 4 weeks before Ramadan	2 time points: 4 weeks before Ramadan, 1 month after Ramadan	No alteration in sleep time during Ramadan
de Toledo (92) 2019 Germany	Cross-sectional	Long-term or prolonged fasting: 4–21 days of Buchinger periodic fasting	Adults *N* = 1,422	Baseline	2 time points: At the baseline and after completing the fasting duration	Enhanced physical and emotional well-being and no feeling of hunger
Hussin (93) 2013 Malaysia	RCT	Fasting and calorie restricted diet: a reduction of 300–500 kcal in daily energy intake combined with 2 days of Muslim Sunnah fasting per week for 3 months	Aging men *N* = 16	Routine diet *N* = 15	12 weeks	Compared to the control, tension, anger, confusion, and total mood disturbance were significantly reduced in the intervention group and vigor was improved
Michalsen (94) 2006 United States	RCT	Periodic fasting: modified fasting period with restricted daily energy intake to maximum 300 kcal	In patients with mild to moderate chronic pain syndromes *N* = 36	Control group (vegetarian meals with 2000 kcal/day) *N* = 19	8 days	A significant mood enhancement at the late study in fasted patients compared to the control. The feeling of hunger was mitigated over the study period for the majority of fasting patients
Kessler (95) 2018 Germany	NRCT	IF (24 h fasting/week): fasting once a week which was repeated every week for 8 weeks, with abstinence from solid food between 00:00 and 23:59 and energy intake restricted to maximum 300 kcal; routine diet on feed days	Healthy volunteers *N* = 22	Control group with routine diet *N* = 14	6 months	No significant difference was found between intervention and control group at week 8 and after 6 months follow-up regarding mental health related outcomes such as positive mood, vigor, depression, tension, fatigue, and anger. Improved depression, anxiety, and HRQoL in the fasting group favor at the month 6 of follow-up
Gabel (96) 2019 United States	Pre-post non-randomized RCT	TRF: *ad libitum* food intake within an 8 h window (10:00–18:00) and fast (18:00–10:00) on a daily basis	Obese adults with BMI 35–40 kg/m^2^ *N* = 23	Baseline 2 weeks with routine diet	12 weeks	No significant changes in the adverse neurological outcomes including dizziness, weakness, fatigue, unhappiness, headache, and irritability
Kleckner (97) 2022 United States	Pre-post non-randomized RCT	TRF: daily 10 h window in accordance with their routine diet and preferences	Post-cancer treatment patients *N* = 36	Baseline	14 days	Improved fatigue and mood
Widhalm (98) 2017 Austria	Pre-post non-randomized RCT	ADF: a complete fasting day and normal food intake on the next day	Healthy overweight or obese individuals *N* = 9	Baseline	12 weeks	An improvement of mental well-being and vitality after 4 weeks from the baseline
Riat (99) 2021 Germany	Explaratory study	RF: fast the entire month of Ramadan from sunrise to sunset	Healthy participants *N* = 34	Baseline (week before RF)	6 time points: week before RF, day1, day14–16, and day27–29 of RF, week after RF, and month after RF	Enhancement in mood status and fatigue mediated by BDNF and cortisol
Alfahadi (100) 2020 Saudi Arabia	Case-control	RF: fasting the entire month of Ramadan for 15–16 h/day	Patients with T2DM *N* = 39	Healthy subjects *N* = 43	2 time points: Between 15th and 22nd day of Ramadan, 2–3 after Ramadan	No effect on fatigue severity when comparing during and post fasting periods. Fatigue severity of patients with T2DM during and after Ramadan was lower compared to the controls

RCT, randomized clinical trial; TRF, time restricted feeding; WC, waist circumference; HRQoL, health-related quality of life; ADF, alternate day fasting; ISI, the insomnia severity index; IF, intermittent fasting; BDNF, brain-derived neurotrophic factor; T2DM, type 2 diabetes mellitus.

In a 3 months trial, intermittent energy restriction (at 25% energy requirement) on fast days and an *ad libitum* diet (non-fast days) were applied to overweight or obese women (70). Compared to the baseline, sleep duration in subjects increased on fast days, and it was more evident at week 2 in comparison with the final week. Sleep duration was also greater on the fast days compared to the feed days in week 2, but not in the final week. As a result, a reduction in physical activity due to more sleep in the early phase of the trial (week 2) was speculated to influence weight loss success with intermittent energy restriction (70). One of the most studied fasting regimens, TRF, has been investigated within different windows of eating time from 4 to 10 h each day and then fasting for the rest of the day. In a cross-sectional study, TRF with an eating window of 8 h had no effect on the sleep quality of elderly adults assessed by the Pittsburgh sleep quality index (PSQI) (81). Similarly, TRF with shorter windows (4–6 h) did not alter sleep time, sleep quality, or insomnia severity over the 8 weeks trial in obese adults, although it resulted in significant weight loss (82).

Macronutrient timing is one of the exogenous factors influencing a normal circadian rhythm (101). It has been shown that carbohydrates are most tolerated and proteins are best utilized in the early active phase (101). Besides, circulating triglycerides (TGs) were lower at midday compared to early morning, implying more efficient uptake by muscles and brown adipose tissue during the active phase (101). TRF with an early feeding phase is very close to the normal eating pattern, as metabolism is most efficacious in the early phase and the consumed food can therefore be best used (101). The evening fast is hence able to inhibit the deleterious effects of a high-fat diet at this time and the subsequent increase in TGs. Besides, the increase in postprandial core body temperature after an extreme carbohydrate intake and the subsequent suppression of melatonin expression can also be hindered by late fasting (101). This can be of help to shift workers when light exposure at night leads to a suppressed release of the sleep hormone melatonin and results in overall sleep deprivation on workdays (102).

The impact of meal timing on sleep and performance was examined in healthy individuals who simulated shift work for four consecutive days (83). In this trial, subjects were randomized into two groups: eating and not eating at night. The former had overnight eating and 12 h fasting during the day (early phase). The latter restricted the eating hours from 7 a.m. to 7 p.m. and had overnight fasting (late-phase). It was shown that sleepiness was increased in both overnight eating and non-eating individuals. However, no significant changes were observed regarding sleep variables such as total sleep time, wake after sleep onset, and sleep onset latency (83). It was also shown that not eating at night ameliorated the performance impairments observed in shift workers allowed to eat at night (83).

Sleep impairments have been an issue for people with T2DM when developing the condition and in its control phase. Studies have shown that there is a causal relationship between sleep disturbances and impaired glucose metabolism, which could be improved when sleep time or quality is improved (103). From animal studies, it has been speculated that TRF may be considered an adjunctive therapy for individuals with diabetes who are more likely to face sleep disturbances (104). TRF with a 9 h window, however, in either an early (8 a.m. to 5 p.m.) or delayed (12 p.m. to 9 p.m.) condition had no effect on sleep duration when compared to the 7 days baseline in men at risk for T2DM, but reduced the mean fasting glucose regardless of the time TRF was commenced (31). Similarly, in patients with diabetes, adherence to a 4 weeks TRF with an 8–10 h/day window (between 10:00 and 19:00) had no effect on mental well-being indicators including sleep quality, duration, disturbance, and daytime sleepiness measured through the PSQI (84). Besides, no significant change was shown for additional indicators, including quality of life, depression, anxiety, and stress in patients (84). Yet, TRF was found to be practical in patients with diabetes, at least to reduce daily energy intake compared to non-adherence days (84).

In a before-after design, the effect of TRF was examined in a sample of healthy individuals and abdominal obese patients (85). Participants reported their health-related quality of life (HRQoL) before and after 3 months of daily feed restriction to 8–9 h. There was an increase in HRQoL and sleep quality, but sleep duration was not altered. The average fasting time and sleep quality at baseline were associated with differences in HRQoL. There was a correlation between sleep quality enhancement, baseline sleep quality, and HRQoL at follow-up. The TRF effect on HRQoL, or sleep quality enhancement, was independent of changes in anthropometry and weight loss (85). Overall, TRF has shown good compliance, and it is practical to adapt to everyday life. Nevertheless, whether the merits of TRF, such as improving performance, sleep disturbances, or HRQoL, outweigh its disadvantages, such as elevated hunger or satiety, needs further investigation.

In obese pre- or postmenopausal women, an ADF approach (600 kcal “fast day”; *ad libitum* diet “non-fast day”) combined with restricted carbohydrate intake to 30% and an equal proportion (35%) of fat and protein was used to examine the effect of weight reduction on sleep pattern (86). Body weight and fat mass decreased during the time of weight reduction (the first 3 months of the study), and the decreases remained throughout the weight maintenance period (the last 3 months of the study). The PSQI score showed low sleep quality at baseline, with no change by months 3 or 6. Compared to the baseline, the insomnia severity index (ISI) and proportion of obese adults with high risk of obstructive sleep apnea indicated no differences by months 3 or 6. Moreover, there were no differences between wake time, bed time, and sleep duration at the three time points (86). Studies have usually evaluated the possible effects of FDs on various health outcomes on an individual basis. In a comparison between a calorie-restricted diet versus an IF diet (both diets had 70% energy requirements), healthy overweight or obese women were randomly assigned to follow one of the diets (27). After 8 weeks of intervention, neither of the groups had an effect on various components of sleep quality measured by the PSQI, quality of life items such as social functioning, fatigue, and health in general using the 36-item short-form health survey (SF-36), or mood status assessed by the DASS (depression, anxiety, and stress scale) (27). Besides, no significant differences in mean changes of the previously mentioned outcomes were observed between CR and IF. Considering the conflicting results of the studies, further research is required to elucidate the effect of intermittent fasting on sleep patterns in different populations.

Regarding sleep patterns, the impact of Ramadan fasting has also been examined in a number of studies. During the month of Ramadan, lifestyle changes such as nocturnal light exposure, interrupted sleep, and eating at night might affect individuals in terms of sleep pattern, alertness, and overall well-being. To exemplify, greater daytime sleepiness was observed during Ramadan fasting (87). The study of eight male volunteers indicated that total sleep time measured in the second week of Ramadan did not differ from that at the baseline (the last week before Ramadan) (88). Besides, diurnal fasting in Ramadan had no effect on sleepiness or alertness evaluated by electroencephalography, while volunteers had a delay in sleep and wake time during the month of Ramadan compared to the baseline (88). In another study, similar results were reported regarding daytime sleepiness using both subjective and objective methods, including the Epworth sleepiness scale (ESS) and the Johns drowsiness scale (JDS), respectively (89). It was also shown that wake time was significantly delayed in the first and second weeks of Ramadan in comparison with the baseline (1 week before Ramadan) (89). Moreover, the study showed a significant change in sleep duration measured through the SenseWear Pro Armband^TM^, and it decreased during Ramadan compared to the baseline (89). In addition to the different methods of sleep assessment used in these studies, lifestyle changes such as an adaptation to delayed work hours during Ramadan or being entertained by TV programs late at night have also been implicated as reasons for the different results observed in the literature regarding the changes in sleep patterns, especially in Muslim countries (105). Using a wearable device (Fitbit Flex 2), sleep time and length were examined in 36 T2DM patients (90). Compared to the after-Ramadan period, daily total sleep time and night sleep time decreased in the middle of Ramadan (90). Sleep deprivation or any disturbance in sleep quality or quantity has been shown to affect nocturnal blood pressure, and if persistent, it might cause hypertension or make it difficult to control the condition (106). Adhering to diurnal Ramadan fasting with plausible lifestyle changes such as interrupted night sleep might therefore affect people with hypertension. A cross-sectional study, however, indicated that Ramadan fasting had no adverse effect on the blood pressure of hypertensive patients in comparison with the after-Ramadan period (91). Besides, no effect of fasting was found on fatigue, sleeping time, or physical activity among the hypertensive participants (91).

When examining health outcomes such as sleep, it’s crucial to acknowledge that self-report measures, like the Pittsburgh sleep quality index (PSQI) or the Epworth sleepiness scale (ESS), are subjective measure tools. While self-reporting empowers subjects, it poses the risk of conveying inaccurate information, especially if individuals struggle with comprehending written content, have visual impairments, or encounter difficulties in physically documenting their responses (107). Moreover, most self-report measures exhibit significant correlations with self-reported mood (108). This linkage could potentially influence responses to a self-report questionnaire, particularly in the absence of concurrent mood measurement and statistical adjustments (108). Caution is, therefore, needed when interpreting the lack of changes observed using a self-reported sleep measure. Although some noncompliance was reported in some studies, such as sleep deprivation or daytime sleepiness, the majority of FDs showed good acceptability and positively influenced participants’ health. FD, as a modifiable lifestyle factor, would therefore be applied as an adjunctive therapy in combination with pharmaceutical treatments. However, further longer trials with larger sample sizes are needed to focus on FDs, mainly TRF and ADF, to first investigate which duration or frequency of these diets is more efficient to confer health benefits related to sleep disturbances. Secondly, whether these diets could be a promising strategy to lessen or hinder the negative health effects of circadian rhythm disturbances such as shift work or other sleep-interrupting conditions like diabetes is also a matter that needs further elucidation.

### Fasting diets and mood (neurological outcomes)

Mood states have been examined across various dimensions of mood swings over time, encompassing, tension or anxiety, anger or hostility, vigor or activity, fatigue or idleness, depression or dejection, and confusion or bafflement (93). Several scales and questionnaires, including the profile of mood states (POMS) (93), Beck’s depression inventory (BDI)-II, and the hospital anxiety and depression scale (99), have been employed to assess mood in these studies. (99). Regarding the effect of FDs on neurological outcomes, quality of life has been another concept being examined using different approaches, for instance social indicators, economic indices, or subjective well-being (SWB) (109). This review focuses on the SWB approach, which focus on happiness and/or satisfaction with life, defining well-being in terms of pleasure attainment and pain avoidance (109). Additionally, the psychological well-being (SWB) approach concentrating on meaning and self-acceptance and defining well-being based on the extent to which a person is fully implementing, is considered (110). In the human studies included in this review, quality of life has predominantly been assessed using various measures, such as fatigue severity scale (99, 100) and SF-36 (98), with the SWB approach being the dominant perspective.

Several animal and human studies have demonstrated the positive effects of fasting on mood improvement (13, 14). Prolonged fasting, used as an adjuvant therapy in the cure of chronic pain syndromes, has been shown to improve mood (111, 112). In a prospective cohort, the effect of Buchinger periodic fasting on the well-being of 1,422 subjects was assessed (92). The fasting duration ranged from 4 to 21 days, and physical and emotional well-being were daily self-reported by participants using numeric rating scales. Fasting for any duration led to a rise in ketone body levels, indicating the metabolic switch from liver-derived glucose to ketones produced in adipose cells. Besides, enhanced physical and emotional well-being and no feeling of hunger in 93% of the subjects supported the accessibility of this periodic fasting (92).

In a clinical trial, 3 months of intermittent fasting (5:2 with a daily reduction of 300–500 kcal from baseline energy intake) in aging men demonstrated that depression status was not significantly changed over the study period (93). When compared to the control group, tension, anger, confusion, and total mood disturbance were significantly reduced in the intervention group, and vigor was improved (93). The effect of 8 days periodic fasting (restricted energy intake to 300 kcal) on mood status was examined in patients with chronic pain syndrome using a self-rating 100 mm visual analog scale (94). At the beginning of the study, mood status was comparable between the two groups; however, towards the end of the study, significant mood enhancements were observed in fasted patients compared to the control. Besides, the feeling of hunger assessed with a standardized Likert scale was mitigated over the study period for the majority of fasting patients (94). An additional study by Kessler et al. (95) reported the effect of IF (24 h/week) on mental health-related outcomes compared to a regular diet in healthy volunteers. At the 6 months follow-up, there was a significant change in HRQoL (a useful indicator of overall health) and the total score of depression and anxiety in favor of the fasting group. No significant difference was found between the intervention and control groups for other mental health-related outcomes such as positive mood, vigor, tension, fatigue, and anger (95).

In other studies, TRF inhibited behavioral changes and reduced neuroinflammatory biomolecules associated with mood regulation in the brain. This indicated that TRF in synchrony with the light-dark cycle can prevent neuroinflammation, leading to healthy mood states despite a disturbed circadian cycle (113). In a human study, however, time-restricted eating with a 16:8 pattern for 12 weeks did not significantly alter adverse neurological outcomes including dizziness, weakness, fatigue, unhappiness, headache, and irritability in obese adults (96). The effect of TRF on mood-related outcomes has not been limited to healthy individuals. In post-cancer treatment patients, preliminary results indicated that TRF with a 10 h window for 14 days successfully improved fatigue and mood (97).

The effect of ADF -being fasted for a complete day and having normal food intake on the next day-on the psychological features of 15 overweight and obese women resulted in improved subjective psychological well-being (using the SF-36 health survey questionnaire) and vitality after a 12 weeks intervention (98). Nonetheless, no effect was observed regarding psychological examinations including concentration, reaction time, and mood (three dimensions of mood: good or bad mood, alertness or tiredness, and calmness or restlessness assessed with the multidimensional mood questionnaire) (98).

Ramadan fasting has most often been associated with mood comfort. The effect of Ramadan fasting on mood states (depression and anxiety), fatigue, and daytime sleepiness was examined in 34 healthy participants (99). BDNF and cortisol, as associating factors with mood status, were measured. The study indicated that BDNF levels were affected by fasting and decreased during the month, but returned to normal levels 1 month after Ramadan (99). Cortisol levels decreased 1 week after Ramadan and reached normal levels 1 month after Ramadan. The changes in BDNF and cortisol were significantly correlated with an improvement in mood status and feelings of fatigue. Moreover, assessing some biological factors such as IL-8 and IGF-1, it was also demonstrated that the observed effects of BDNF and cortisol could be mediated by these factors (99). The association of IL-8 and IGF-1 with mood status has been reported in other studies (114, 115). The significant correlation observed between BDNF, cortisol, and the mediators (IL-8 and IGF-1) with body composition parameters confirmed the beneficial effect of RF during this period (99). Regarding the inflammatory cytokines such as IL-8 and IGF-1, it’s worth noting that fasting diets such as IF have demonstrated immunomodulatory effects, partly mediated by the gut microorganisms (116). The composition of the gut microbiota has been shown to be influenced by diet and feeding patterns (117). Inappropriate dietary intakes may lead to an undesirable shift in microbial composition, referred to as dysbiosis, resulting in increased permeability of the intestinal mucosa. This, in turn, facilitates the crossing of bacteria through the intestinal barrier, binding to circulating macrophages and monocytes, ultimately contributing to an elevated inflammatory state through the secretion of proinflammatory cytokines like TNF-α, IL-1, and IL-6 (81). Gut commensal bacteria and their metabolites have the potential to exert both pro- and anti-inflammatory responses by regulating T cell differentiation and immune responses within the gut. Fasting diets have demonstrated the ability to increase gut microbial diversity and richness while promoting a shift in their composition and metabolic pathways towards a healthier gut (116). The intestinal microbiota is involved in modulating and regulating the formation of the host’s immune system (118). Studies have indicated that TRF may reduce plasma levels of inflammatory cytokines such as TNF-α, IL-6, and IL-1b along with an increase in adiponectin levels (119). Higher concentrations of proinflammatory cytokine IL-6 and lower adiponectin levels were associated with sleep disruptions and diseases (120, 121). Growing evidence suggests a reciprocal relationship between diet and sleep (122). Research have shown that sleep restriction can decrease adiponectin levels in healthy individuals (123), whereas IF may elevate the levels of this cytokine (116). In another study, Ramadan fasting had no effect on fatigue severity in either the type 2 DM patients or the control group (100). Yet, the fatigue severity of patients with T2DM during and after Ramadan was lower compared to the controls (100).

Studying different populations, the effectiveness of FDs in improving mood-related outcomes has been demonstrated in the short term. Yet, further studies are needed, firstly to examine the potential beneficial effects of FDs on mood over longer periods and, secondly, to investigate further whether FDs are healthy and could be used as adjunctive therapy in vulnerable subjects with specific diseases like cancer.

### Mechanisms underlying the interconnection between fasting, eating behaviors, sleep, brain and mental health

The eating window, e.g., morning or evening, in fasting diet interventions might be one of the possible factors influencing food-related behaviors. In a 4 days crossover, isocaloric controlled trial in 11 overweight subjects, Ravussin et al. (30) reported that evening TRF (18 h: fasting from 2 p.m. to 8 a.m.) compared to the control group (12 h: fasting from 8 p.m. to 8 a.m.) appeared to significantly decrease swings in hunger and desire to eat and increase fullness during the day, as well as reducing fasting ghrelin and increasing PYY hormone levels in the evening. Reduced desire to eat and satiety hormone PYY, along with unchanged appetite and ghrelin levels, were revealed by Sutton et al. (32) following 5 weeks of evening daily fasting for 18 h (from 3 p.m. to 8 a.m.) compared to daily fasting for 12 h (8 a.m. to 8 p.m.) with matching calorie intake in prediabetic adults. One of the issues emerging from these findings is that the food intake window of TRF is compatible with circadian rhythms in metabolism through food consumption in the morning and food abstinence in the afternoon onwards, and it might be beneficial for hunger, appetite, and mediating increased compliance (32, 83, 124). Furthermore, improved metabolic health has been reported by evening fasting (124, 125). However, a 7 days TRF intervention (eating window: 8 a.m.–5 p.m.) did not alter eating behaviors or hunger-satiety hormones compared to a TRF intervention (eating window: 12 p.m.–9 p.m.) (31). With the small size and short-term duration of these investigations, caution must be applied when interpreting the results.

Several experimental studies have uncovered that altered feeding-fasting cycles play a notable role in controlling signaling pathways involved in the circadian clock (126). Enhanced expression of the circadian genes, including circadian locomotor output cycles protein kaput (Clock) and brain and muscle ARNT-like 1 (Bmal1), by the increase in sirtuin 1 (Sirt1) expression, has been found following fasting for 16 h a day for 25 days in healthy subjects (127). The TRF and CR diets affect different regions of the circadian clock. The suprachiasmatic nucleus (SCN), or primary circadian clock, is mostly affected by the CR diet, and both primary and peripheral circadian clocks are influenced by TRF. The peripheral circadian clocks are the parts controlled by eating timing, sleeping, and other lifestyle factors (128). Food deprivation can cause changes in peripheral clocks mediated by switching from nocturnal to diurnal eating (129). However, the effects of different fasting diets on circadian rhythm modifications related to appetite regulation need to be clarified and addressed in further studies.

A recent review summarized the studies that investigated the effect of the CR diet and various time-restricted fasting diets on gut hormones. The study showed that a CR diet is associated with increased ghrelin as an orexigenic hormone and reduced satiety hormones including leptin, insulin, glucagon-like peptide-1 (GLP-1), peptide YY (PYY), and cholecystokinin (CCK). Attenuated satiety signals, including leptin, GLP-1, and PYY, are reported following TRF (17). There are inconsistent findings regarding insulin following TRF. Based on limited short-term studies, decreased or unchanged ghrelin through the hypothalamic melanocortin system has been found by TRF (17).

Ghrelin is a gut-derived hormone that has a cephalic phase of secretion related to orosensory stimulation and experienced meal pleasure (17). The sustained rise after the CR diet has been linked to the “grazing” food pattern (17, 130). Lamont et al. (131) suggested that due to the expression of ghrelin receptors in the suprachiasmatic nucleus (SCN) and master clock, ghrelin may play a role in modulation of the circadian system during TRF. Thus, it seems that the hunger-satiety balance following a CR diet probably inclines toward hunger, which is less probable to occur after TRF diets. Dissimilar to the CR diet, improved calorie balance and mitigated hyperphagia following TRF have been mediated by ghrelin and leptin alterations through the hypothalamic melanocortin system (128). However, currently there is a gap regarding the alterations of several gut peptides related to satiety regulation by fasting diets. In addition, the short-term and highly different feeding and fasting patterns of the present studies make it difficult to reach definitive conclusions. Further studies are needed to clarify more details in this area.

Eating behaviors and fasting diet compliance might be influenced by adverse effects of FD, including gastrointestinal and neurological symptoms, which have not been taken into consideration. A 6 months ADF with a low-CHO diet resulted in an increase in constipation, bad breath, and dry mouth (26, 36). Vomiting, increased thirst, headaches, and diarrhea have also been observed in response to a 5 weeks TRF (eating window: 8 a.m. to 3 p.m. and 18 h of fasting) (32). Feeling lightheaded, constipated, and irritable on fasting days were found during a 21 days ADF among non-obese individuals (42). The negative effects of fasting diets with regard to eating behaviors are necessary to be considered in future studies.

When we eat, glycogen is stored in the liver and can be utilized as the major energy source for our body cells. In individuals who are not exercising, glycogen stores run out after 10–14 h, and free fatty acids, released by adipose tissue, are converted to ketone bodies in the liver to supply energy (132). When exercising, glycogen stores are more rapidly depleted, and the metabolic switch from carbohydrates and glucose to free fatty acids is accelerated. The metabolic shift during fasting affects signaling pathways to reinforce cellular stress resistance and prepare the conditions for subsequent cellular structural and functional plasticity that occurs during the feeding state (132). Here, we discuss intermittent fasting approaches as fine examples of the metabolic shift during which the molecular and cellular adaptations of neurons in association with mood regulation occur.

**Figure 1:**
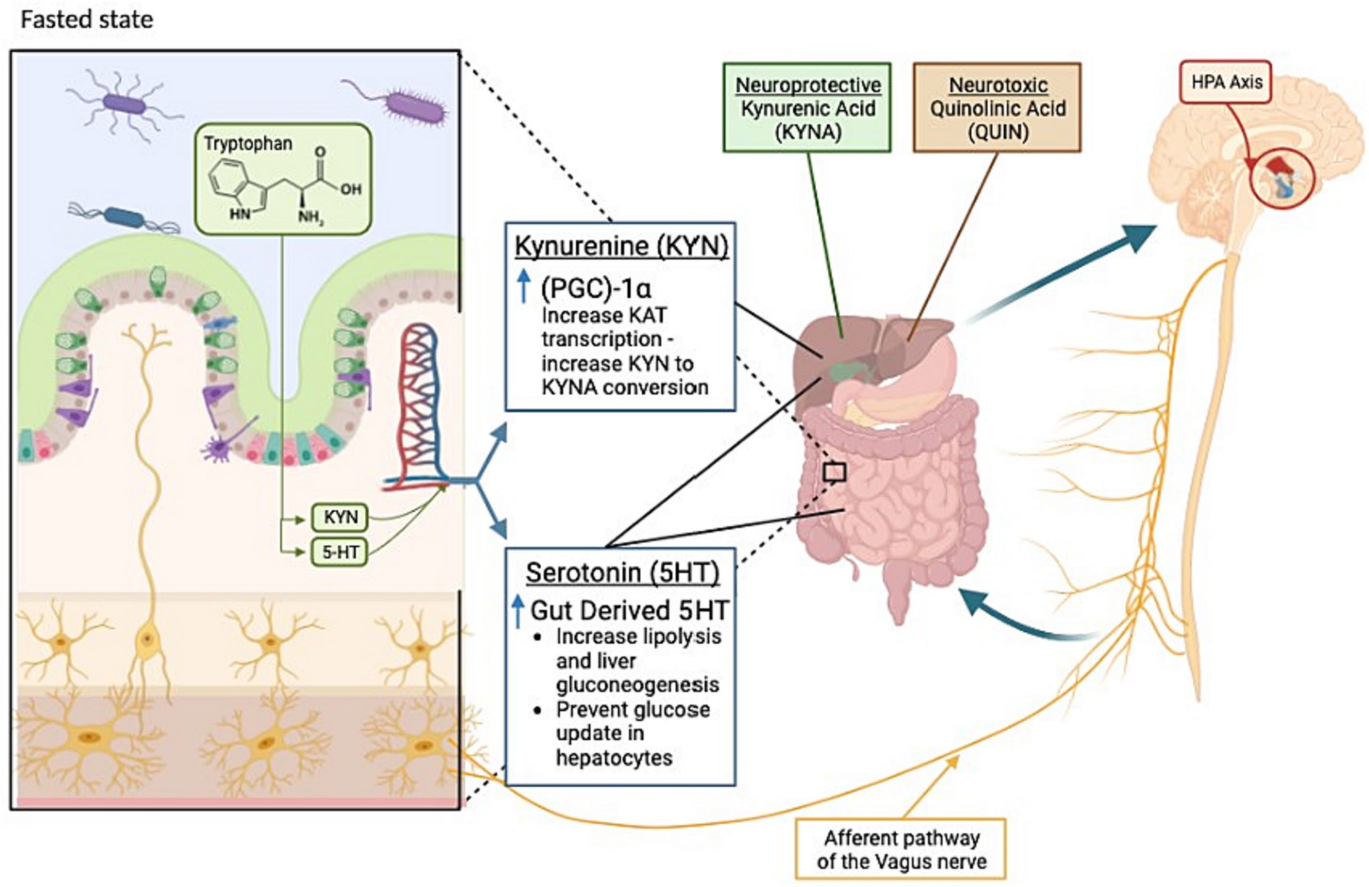
Key changes occurring in the gut-brain axis during fasting states. During a fasted state, the gut (left) and brain (right) display changes as indicated. Kynurenine metabolism products kynurenic acid and quinolinic acid are identified as neuroprotective or neurotoxic, respectively. Highlighted by the red circle, the hypothalamic-pituitary-adrenal (HPA) axis also plays a role in the bi-directional communication between the gut and brain during fasting. Kyn, Kynurenine; KYNA, Kynurenic acid; 5-HT, Serotonin; KAT, Lysine acetyltransferase; QUIN, Quinolinic acid; PGC-1α, Peroxisome proliferator-activated receptor gamma coactivator-1 alpha.

Overall, fasting benefits mental health by affecting neuroplasticity, neurogenesis, bioenergetics, and stress resistance in the nervous system. Mood comfort during fasting has been discussed as an evolutionary adaptation for survival and the search for food (111). While not fully characterized, the currently known effects of fasting on the gut-brain axis can be found in Figure 1. The neurobiology of mood improvement during periodic fasting has been discussed through several mechanisms to be discussed below (111).

The two-way communication between the gut and the brain has been of interest when examining the effects of fasting diets on mood. It has been shown that gut-brain cross-talk can affect the brain function, linking emotional and cognitive centers of the brain with peripheral control and function of the gut (133). In the gut-brain axis, the bidirectional communication between the gut bacteria and the brain plays a vital role in maintaining brain health (134). Several systems are involved to assure the efficient functioning of the gut microbiota-brain axis including, the central nervous system, the enteric nervous system, the sympathetic and parasympathetic branches of the autonomic nervous system, and neuroendocrine and neuroimmune systems (135). The microbiota has the potential to affect neuronal function directly or indirectly through neurotransmitters, vitamins, and microbial metabolites such as short-chain fatty acids (135). It’s difficult to understand the mechanism by which these metabolites affect brain function because intestinal-mucosal barrier and blood-brain barrier hinder a direct access to the brain (133). It’s suggested that the microbiota may send signals to the brain by activating afferent sensory neurons of the vagus nerve via neuroimmune and neuroendocrine pathways, affecting the production of precursors, neurotransmitters, and metabolites that are associated with mood and behavior (133, 134). The prevalent gastrointestinal ailments observed among patients with anxiety and depression disorders imply a mutual interaction between gut activities and brain function (136). Intermittent fasting has been shown to modulate the gut microbial composition and their metabolic activities towards an increased production of neurotransmitters and active metabolites. It was indicated that germ-free mice had a higher level of circulating tryptophan and low levels of hippocampal serotonin, which conversely increased when introducing tryptophan-metabolizing bacteria into the gut, along with a mitigation in anxiety-like behavior (137).

One key metabolite highlighted is that of the essential amino acid tryptophan and its breakdown products, kynurenine (Kyn) and serotonin. As tryptophan is unable to be produced in the body, its metabolism is tightly regulated, and its utilization is altered during fasting (112).

Kyn is the main metabolite produced, with 90% of tryptophan metabolism being utilized for Kyn production (138). Kyn is subsequently metabolized into two different streams: quinolinic acid (QA) and kynurenic acid (KYNA). Traditionally, QA is viewed as neurotoxic and utilized instead for the creation of NAD^+^, whereas KYNA is viewed as neuroprotective due to its interaction with N-methyl-D-aspartate (NMDA) receptors (139). The transition from Kyn to KYNA is governed, in part, by peroxisome proliferator-activated receptor gamma coactivator 1-alpha (PGC1α). Fasting has been shown to increase the expression of PGC1α. This increase results in a higher allocation of Kyn for the production of KYNA, resulting in an increase in neuroprotection (140). The second tryptophan metabolite, serotonin (5-HT), has two distinct types: gut-derived serotonin (GDS) and brain-derived serotonin (BDS), each with their own areas of effectiveness. GDS constitutes 90% of 5-HT (138). Upregulation of GDS production has been shown to increase during fasting. This increase in GDS results in both lipolysis and liver gluconeogenesis, as well as having an impact on hepatocyte glucose reuptake (141). GDS has also been implicated in the development of the enteric nervous system (139) and subsequent communication with the central nervous system (142). BDS functions as a neurotransmitter and is tied to appetite (143). Fasting has shown the downregulation of BDS transporters and the modulation of central serotonin (112). Also having an effect on BDS, brain-derived neurotrophic factor (BDNF) improves the survival and differentiation of 5-HT neurons (144). A recent study by Elesawy et al. (145) paired an increase in BDNF (and subsequent effects on serotonin) with a decrease in depression and anxiety in rats subjected to an intermittent fasting diet for 3 months. In healthy subjects, the levels of BDNF and serotonin were elevated during Ramadan fasting (146). BDNF and serotonin are both associated with the ability to promote the growth and maturation of neurons involved in mood disorders such as depression and anxiety (144). BDNF also has positive impacts on learning and memory, can stimulate neurogenesis, and enhances mood (112).

In addition to BDNF, other neuropeptides, including neuropeptide Y and orexins, have been increased by fasting and were associated with mood enhancements (147). Neuropeptide Y has shown a pain-relieving effect through the reduction of spinal neuron activity and behavioral signs of inflammatory and neuropathic pain in rodents (148). Orexins have shown anti-depressive effects through extended cell proliferation in the hippocampus (149). Moreover, the release of endorphin hormones is associated with mood improvement in humans (150). In a rat study, brain endorphin levels increased 5-fold after 24 h fasting and were 2-fold higher than those of fed rats after 48 h fasting (151).

It has been indicated that the increased number of neurotrophic factors and protein chaperones following cellular stress might lead to hyperactivity and dysregulation of the hypothalamic-pituitary axis (HPA) in depressed patients (152). However, fasting induces a cellular stress response that can positively affect HPA through the expression of neurotrophic factors and protein chaperones in brain cells and result in mood improvement (94). Cellular stress has been suggested to include a decrease in cerebral glucose, hunger during the fasting state, and reduced insulin and leptin levels (111) although no association between leptin and mood improvement has been found in periodic fasting (152).

Fasting has been discussed to induce the production of ketone bodies that might contribute to mood enhancement and pain relief and confer beneficial neurobiological effects against hypoglycemia and neuronal injuries (112). Ketone bodies have shown neuroprotective and anticonvulsant effects similar to those of CR and ketogenic diets (153). The anticonvulsant effect has been mainly conferred by acetone and acetoacetate (153). The neuroprotective effect, however, has mainly been attributed to-hydroxybutyrate and, to a lesser extent, acetoacetate (153). Mitochondria is the core target for these ketones to confer their effects through a series of activities. In brief, ketone bodies improve mitochondrial respiration, prevent neuronal injury, reduce oxidative stress, and increase adenosine triphosphate (ATP) synthesis. The improved mitochondrial function results in opening the mitochondrial permeability transition pore. As a consequence, cytochrome c release into the cytoplasm of neurons and the subsequent apoptotic cascade are inhibited (153). Moreover, −hydroxybutyrate stimulates the activation of the cytoplasmic transcription factor NF-B in neurons, which then relocates to the nucleus and provokes BDNF expression, thereby tying back to serotonin and the tryptophan pathway, conferring beneficial effects on the nervous system (132).

The circadian system plays a central role in regulating the sleep/wake cycle, determining the timing of sleep onset and offset (154). Furthermore, the circadian phase during which sleep occurs influences its duration, continuity, and structure (154). Light exposure serves as the primary synchronizer for the central clock in the SCN of the hypothalamus, which represses melatonin synthesis by the pineal gland. Artificial light exposure at night can disrupt the SCN and melatonin rhythm (154). Melatonin, responsible for controlling thermoregulatory processes, induces a drop in core body temperature upon release, leading to the onset of evening sleepiness and reduced melatonin level in the morning, contributing to awakening (154).

The two-process model, incorporating a circadian process influencing wakefulness and a sleep-promoting process that intensifies during wakefulness, has been employed to simulate the sleep/wake cycle (155). The SCN regulates the circadian rhythm of body temperature, a key synchronizer of clocks in peripheral tissues (154). Daily temperature oscillations can be eliminated by thermostat use, potentially impacting certain circadian rhythms (101). Apart from temperature mechanisms, the SCN influences peripheral tissue clocks through neural signals transmitted via the autonomic nervous system and the timely secretion of signaling factors such as prokineticin 2 (101). Hypothalamic-pituitary-peripheral organ axes play a crucial role in hormonal regulation of the circadian system (101).

Some behaviors such as sleep/wake patterns, feeding/fasting cycles, and mealtimes, have the potential to disrupt the circadian cycle. Sleep and circadian rhythm disruptions are correlated. Sleep and circadian rhythm disruptions are interconnected, leading to common metabolic dysfunctions such as impaired glucose tolerance, reduced insulin sensitivity, increased inflammatory biomarkers, elevated arterial pressure, and decreased energy expenditure, which results in weight gain (156). This may be attributed to the desynchrony of circadian rhythm concerning actual sleeping and eating times, notably evident in modern societies where adapting to unhealthy dietary patterns and sleeping habits has been associated with mental health and sleep disorders (156).

Chronic disruption of circadian rhythm may also explain the association between mental health and metabolic diseases such as obesity, diabetes, and cardiovascular diseases (157). Diet plays a significant role, as evidenced by the emerging discipline of “nutrition psychiatry” focusing on the association between dietary factors, eating habits, mental diseases, and sleep disorders (158). TRF has been implemented in daily life conditions to potentially alleviate symptoms of sleep deprivation and counteract unhealthy lifestyle behaviors such as night eating or shift working, which disrupt circadian cycle (101). Studies have revealed the beneficial effects of TRF, especially when feeding time is early or in the middle of the day, in regulating glucose and lipid metabolism and improving insulin sensitivity. This is achieved by directly regulating the blood glucose through hormones such as, insulin and glucagon, among others (101), further enhanced by the expression of adiponectin, sensitizing the adipose tissue to insulin and leptin, and mimicking the effect of insulin monotherapy (158).

## Conclusion

Utilization of fasting diets to alter mood continue to have poorly understood effects and treatment options. Currently, it is not fully understood which fasting option provides the most beneficial effects on groups and even less on individuals nor are there consistent trials assessing the effects of fasting diets in a comparable manner. Few short and long-term studies work within an isocaloric/isonitrogenous framework making it more difficult to assess the effectiveness of various fasting regimes. Similarly, baseline assessments and comparable populations are limited in many dietetic studies.

Nevertheless, fasting diets have been shown to have positive short-term effects in relation to caloric restriction diets. The use of fasting diets may provide a new approach for conditions linked to disease co-morbidities or challenges associated with eating behaviors. Altering eating behaviors can have lasting effects on physiological parameters. An examination of the various fasting options and how they impact disease patterns is also merited.

Current evidence suggests that fasting diets, coupled with adequate sleep, and mealtimes synchronized with the circadian clock, contribute to improved metabolic control. To strengthen our understanding of the interplay between fasting diets, circadian rhythm, and sleep in the general population, additional comprehensive human studies are warranted to complement the existing research.

Consideration of eating behaviors, physiological drivers of diet and appetite, and biological sex effects all need to be examined in greater detail. This would be a first step towards individual analysis. But still, circadian rhythms are neglected. Individuals are changing, and we do not know how fast they are changing. This would be real future research (159). However, understanding the impacts of various fasting regimes may provide new insights into the gut-brain axis and offer new treatment avenues for those with resistant anxiety and depression. As research into this field continues to expand, addressing the knowledge gaps in this research will allow us to elucidate the overall impact that timing of nutritional intake can have on overall health.

## Author contributions

EH: Conceptualization, Writing – original draft, Writing – review & editing. AA: Conceptualization, Writing – original draft, Writing – review & editing. JJ: Conceptualization, Writing – original draft, Writing – review & editing. DG: Conceptualization, Writing – original draft, Writing – review & editing. GA: Conceptualization, Writing – original draft, Writing – review & editing. NB: Conceptualization, Writing – original draft, Writing – review & editing. KT: Conceptualization, Writing – original draft, Writing – review & editing. WS: Conceptualization, Writing – original draft, Writing – review & editing. ZM: Conceptualization, Writing – original draft, Writing – review & editing.
